# Pictorial review of the pulmonary vasculature: from arteries to veins

**DOI:** 10.1007/s13244-018-0659-5

**Published:** 2018-10-31

**Authors:** Thomas J. Marini, Kevin He, Susan K. Hobbs, Katherine Kaproth-Joslin

**Affiliations:** Department of Imaging Sciences, University of Rochester, Rochester, NY USA

**Keywords:** Pulmonary artery, Pulmonary veins, Bronchial arteries, Pulmonary medicine, Diagnostic imaging

## Abstract

**Abstract:**

Pathology of the pulmonary vasculature involves an impressive array of both congenital and acquired conditions. While some of these disorders are benign, disruption of the pulmonary vasculature is often incompatible with life, making these conditions critical to identify on imaging. Many reviews of pulmonary vascular pathology approach the pulmonary arteries, pulmonary veins and bronchial arteries as individual topics. The goal of this review is to provide an integrated overview of the high-yield features of all major disorders of the pulmonary vasculature. This approach provides a more cohesive and comprehensive conceptualisation of respiratory pathology. In this review, we present both the salient clinical and imaging features of congenital and acquired disorders of the pulmonary vasculature, to assist the radiologist in identifying pathology and forming a robust differential diagnosis tailored to the presenting patient.

**Teaching Points:**

• *Abnormalities of the pulmonary vasculature are both congenital and acquired.*

• *Pathology of a single pulmonary vascular territory often affects the entire pulmonary vasculature.*

• *Anomalous pulmonary venous flow is named as a function of its location and severity.*

• *Bronchial arteries often undergo dilatation secondary to cardio-respiratory pathology.*

## Introduction

**Fig. 1:**
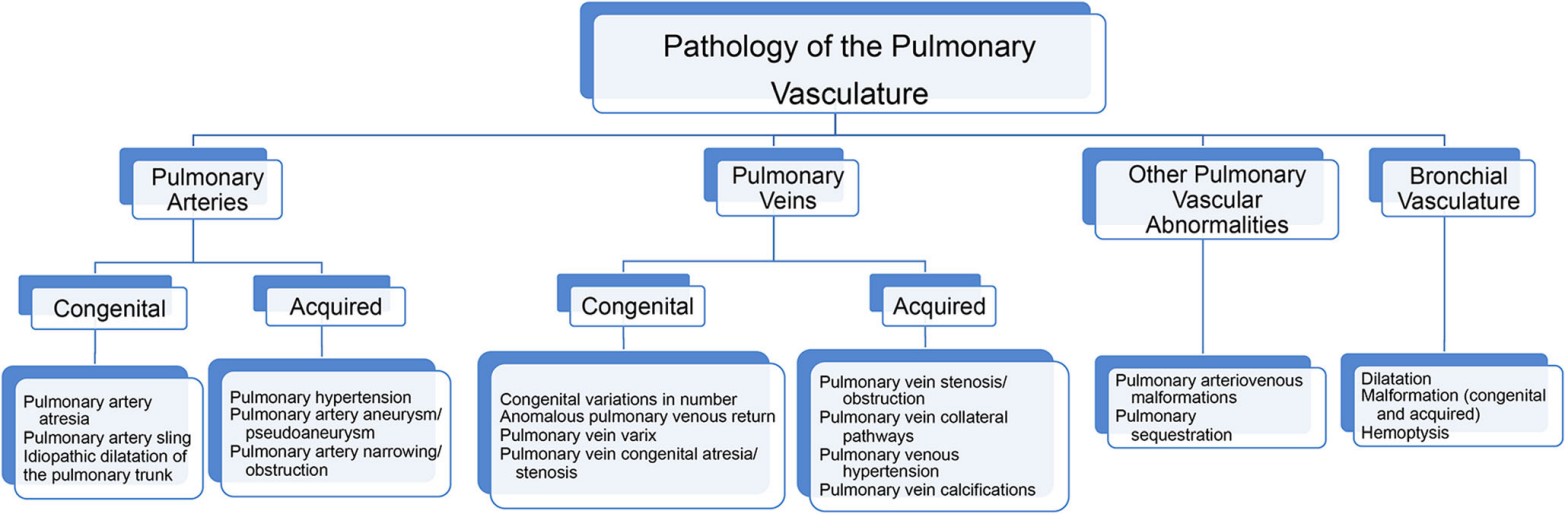
Our conceptual organisation of pulmonary vascular pathology

Disorders of pulmonary vasculature encompass a wide-ranging spectrum of pathology. Although many of these conditions are rare, efficient and accurate diagnosis are essential as many of these disorders are life-threatening. In this pictorial review, we present a guide to disorders of the pulmonary vasculature, covering the congenital and acquired conditions of the pulmonary arteries, pulmonary veins and bronchial arteries. The organisational framework we have adopted for pedagogical purposes can be seen in Fig. 1. Unfortunately, pathology is not so neatly delineated and is often intertwined with other pulmonary systems, as a disruption in any one of these vascular territories can ripple throughout the entire pulmonary vascular system.

## Overview of imaging of the pulmonary vasculature

Multiple imaging modalities are available to assist in the evaluation of the pulmonary vasculature including chest radiography, ultrasonography, magnetic resonance imaging (MRI) and computed tomography (CT). As a general rule, a chest radiograph is limited in its assessment potential for these conditions [1, 2]. In infants and children with suspected congenital anomalies, ultrasound may be the first line choice with MRI as a useful follow-up study to clarify anatomy and follow conditions as it does not expose the patient to ionising radiation [3]. CT remains the “gold standard” for evaluating most vascular pathology due to its ability to clearly elucidate anatomical detail and is often the preferred imaging modality to get a detailed look at complex pulmonary vascular anatomy, at least on initial assessment.

Although pulmonary arteries and veins are commonly imaged without the assistance of electrocardiographically (ECG) gated imaging (MR or CT), in certain circumstances cardiac gating can improve the visualisation of the central pulmonary vasculature. For example, ECG-gating assists in the detailed imaging of the pulmonary vein ostia that is required in the setting of pulmonary vein mapping for pre-ablation imaging and in pre-procedure pulmonic valve replacement planning [2]. Imaging of the pulmonary vasculature is improved using a dedicated angiographic technique in which the timing of the contrast bolus is targeted to the vessel or structure of interest. This is commonly achieved using either a test bolus to determine peak enhancement or a bolus-tracking method to determine when the level of enhancement begins to increase in a structure of interest. For imaging of pulmonary arteries, the main pulmonary artery will be the target structure and for imaging of the pulmonary veins, the left atrium will be the target structure. A contrast flow rate of at least 3 cc/s is recommended to reduce the risk of insufficient enhancement of the target structure. The volume of contrast and duration of scan time needed for imaging will depend on the type of scanner used (e.g. dual source or 16/64/256-slice multidetector CT), with faster scanners decreasing scan time and amount of contrast volume needed [4].

Imaging of the bronchial arteries is often difficult due to their small size and tortuous course. Techniques to improve enhancement of these vessels includes contrast administration targeted to the arterial/aortic phase of imaging to optimise enhancement and three-dimensional volumetric or multiplanar imaging reconstructions to optimise the plane of imaging [5].

## Pulmonary artery abnormalities

### Congenital disorders of the pulmonary artery

#### Pulmonary artery atresia

**Fig. 2:**
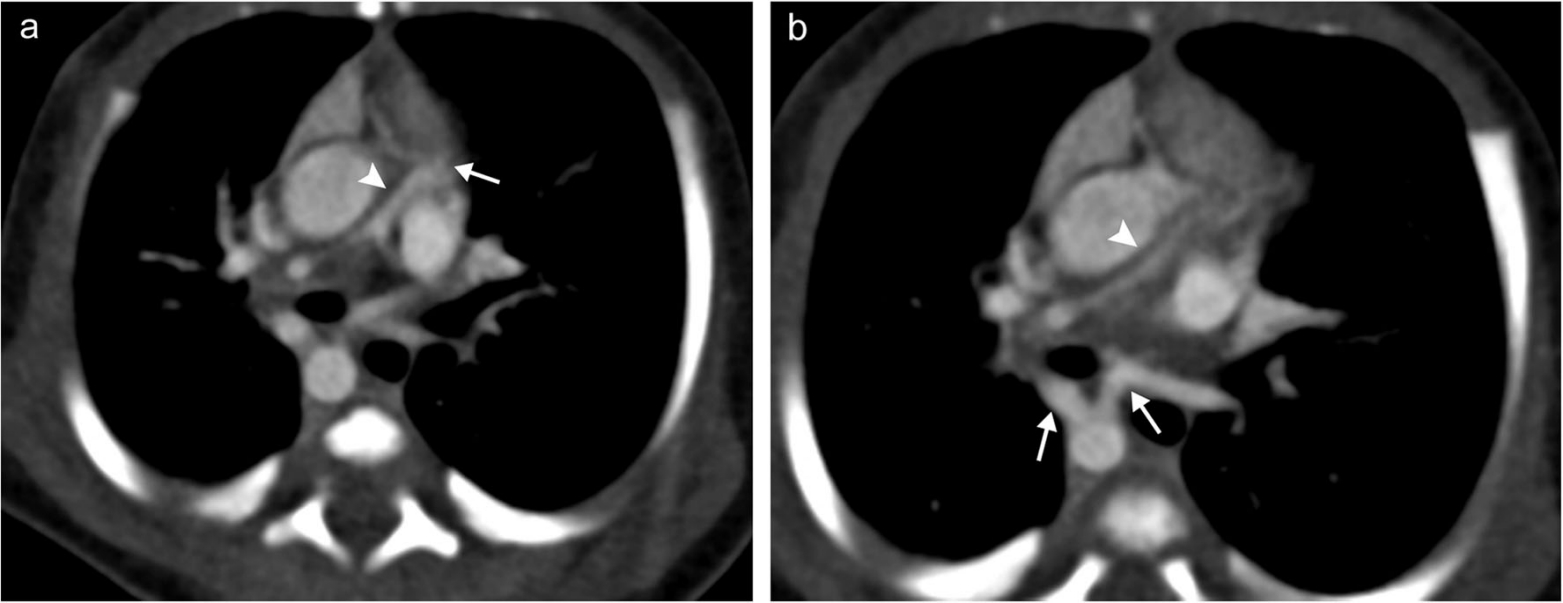
Pulmonary artery atresia. **a** Axial CT image demonstrating abrupt termination of the main pulmonary artery (*white arrow*) in a patient with tetralogy of Fallot with a small hypoplastic distal pulmonary artery (*white arrowhead*). **b** Bronchial artery dilatation off the descending aorta occurs to feed the pulmonary vasculature (*white arrows*) with the hypoplastic distal pulmonary artery again demonstrated (*white arrowhead*)

In rare circumstances, the pulmonary artery fails to fully develop. This condition, called pulmonary artery atresia, is also known as proximal interruption of the pulmonary artery as the distal portion of the pulmonary artery located within the lung is typically intact and perfused secondary to collateral flow from aortopulmonary collateral (APC) vessels. Right pulmonary artery atresia usually occurs in the absence of other congenital abnormalities, while left pulmonary artery atresia often occurs in conjunction with other congenital cardiac conditions, such as tetralogy of Fallot [6]. Most patients with this condition are symptomatic with a combination of dyspnoea, recurrent pulmonary infections and haemorrhage. CT or MRI will show an absence or hypoplasia of the affected artery proximally (Fig. 2). Additional findings include hypoplasia of the affected lung, serration and thickening of the pleura, and APC vessel formation [1].

#### Pulmonary artery sling

**Fig. 3:**
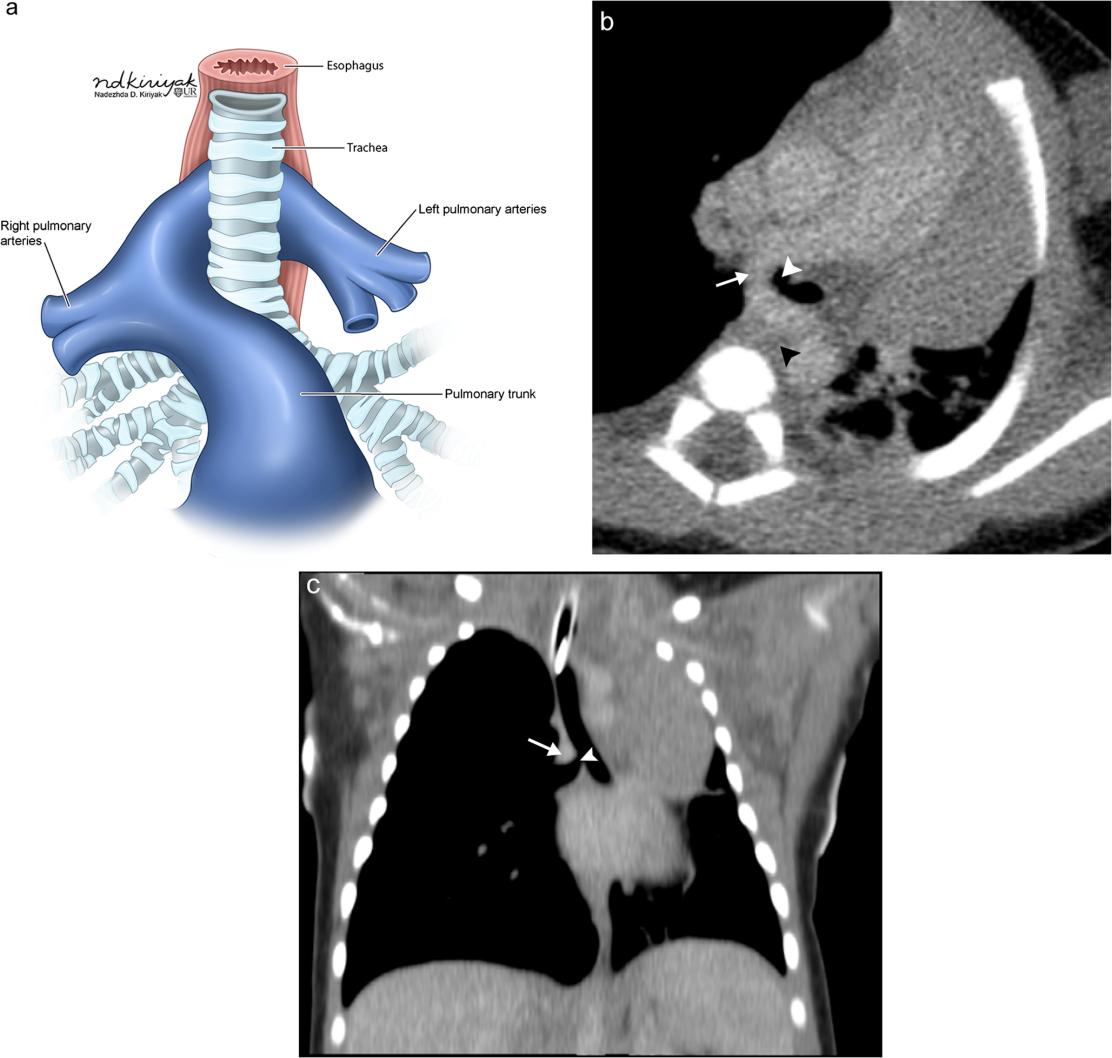
Pulmonary artery sling. Schematic (**a**) and axial contrast-enhanced CT image (**b**) show the left pulmonary artery (*white arrow*) arising from the right pulmonary artery, passing between the oesophagus (*black arrowhead*) and right mainstem bronchus (*white arrowhead*). **c** Coronal maximal intensity projection CT image shows compression of the proximal right mainstem bronchus (*white arrowhead*) by the aberrant left pulmonary artery (*white arrow*)

Developmental failure of the left sixth aortic arch may lead to a pulmonary artery sling, an aberrant origin of the left pulmonary artery arising from the right pulmonary artery, which courses between the trachea/right mainstem bronchus and oesophagus (Fig. 3). This can lead to compression/focal stenosis of the airway and subsequent air trapping and atelectasis, although many patients may be asymptomatic when there is minimal airway compression [1, 7]. While this finding is easily seen on CT and MRI, it may be first detected on plain film imaging as a left-sided deviation of the trachea and mediastinum and on fluoroscopic oesophageal imaging as anterior indentation of the oesophagus.

#### Idiopathic dilatation of the pulmonary trunk

**Fig. 4:**
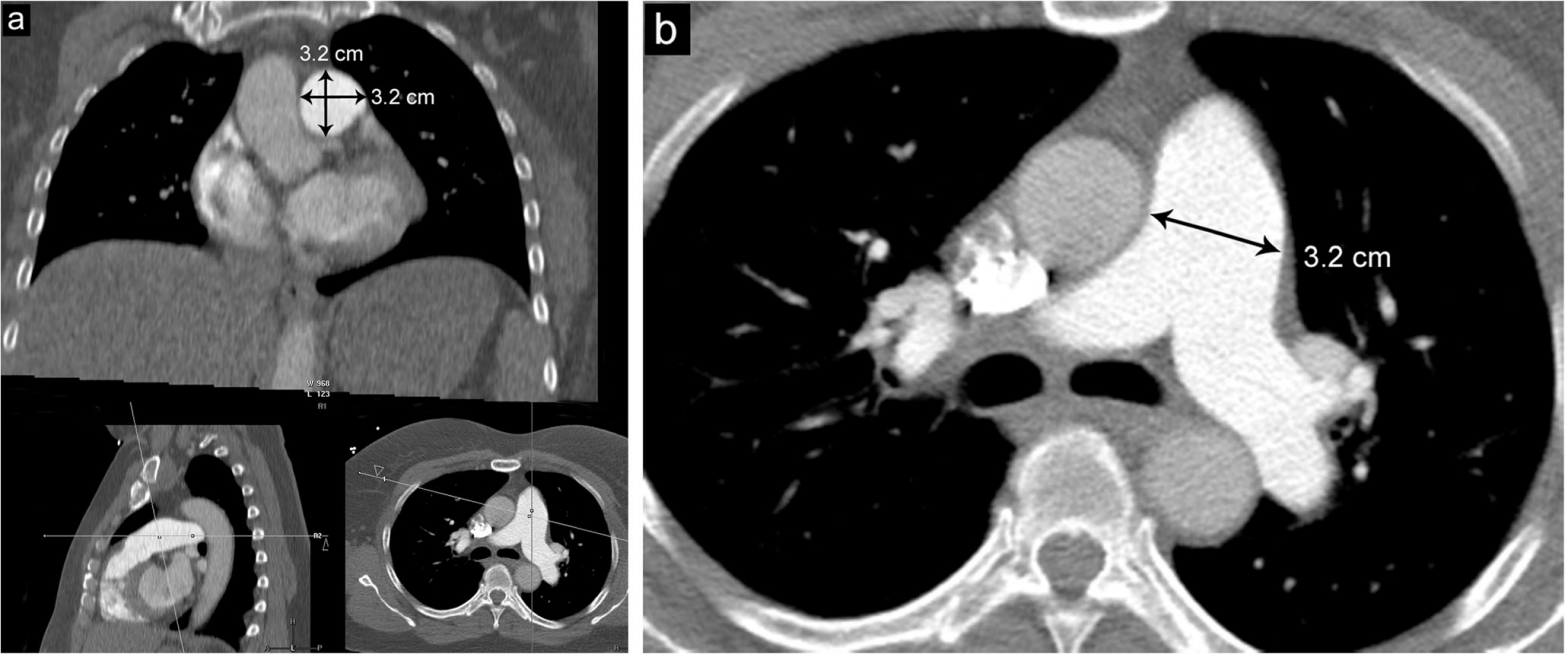
Pulmonary artery measurement. **a** CT imaging demonstrating double oblique measurement technique through the main pulmonary artery. **b** Axial CT image demonstrating transverse measurement through the main pulmonary artery at the level of the right pulmonary artery bifurcation

This is a diagnosis of exclusion in which there is a congenitally dilated pulmonary trunk with normal pressures in the absence of other cardiopulmonary disease. Patients with this condition are, by definition, asymptomatic although close follow-up is recommended [1, 8]. The most accurate method to obtain the main pulmonary artery diameter is to use reformatting software to perform a double oblique measurement of the pulmonary artery at its largest diameter; alternatively, if this technique is not available, the transverse axial diameter of the main pulmonary artery can be measured at the level of the bifurcation of the right pulmonary artery (Fig. 4) [9].

### Acquired disorders of the pulmonary artery

#### Pulmonary hypertension

**Fig. 5:**
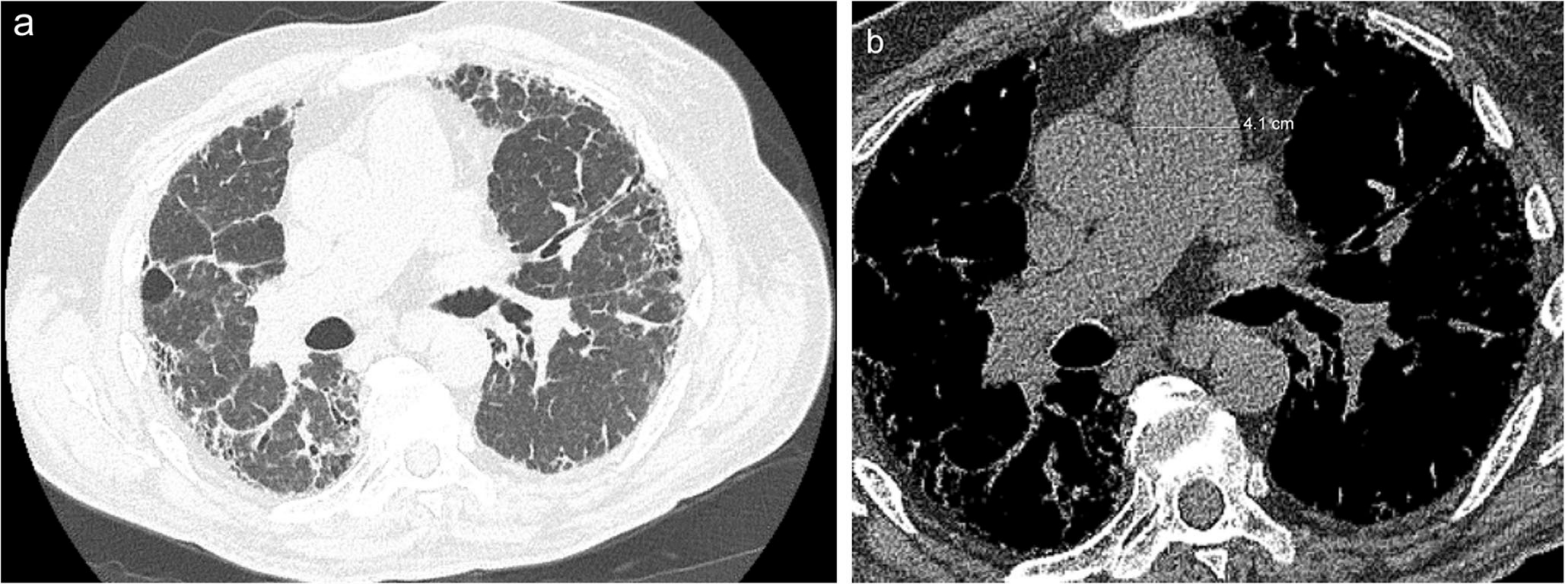
Pulmonary hypertension in a patient with idiopathic pulmonary fibrosis. **a** Axial CT image (lung window) shows basal and peripheral predominant fibrotic disease with reticulation and honeycombing extending to the subpleural surface, as well as traction bronchiectasis, consistent with usual interstitial pneumonia pattern of fibrosis in a patient with idiopathic pulmonary fibrosis. **b** Axial CT image shows dilatation of the main pulmonary artery, measuring up to 4.1 cm in maximal diameter

**Fig. 6:**
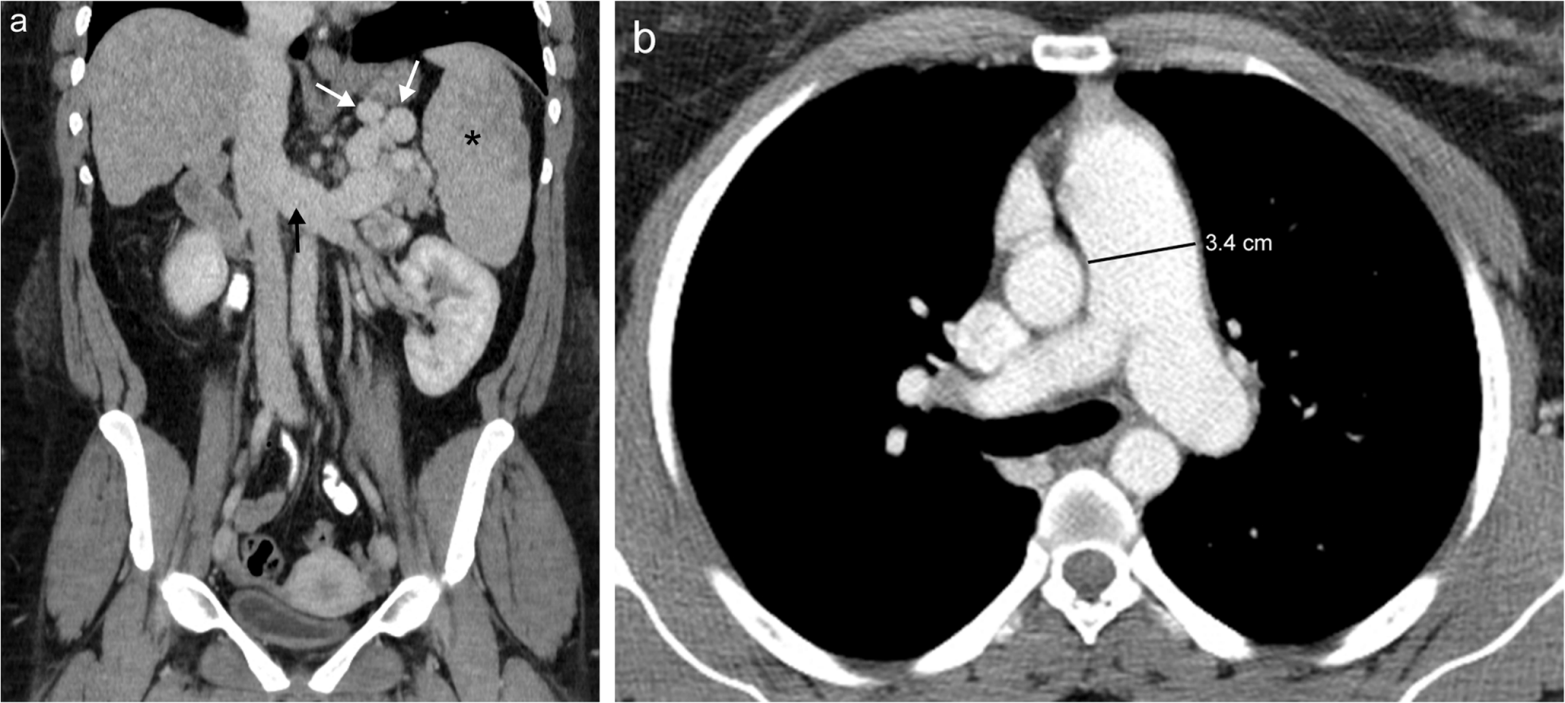
Pulmonary hypertension in a patient with liver disease. **a** Coronal contrast-enhanced CT image shows sequela of portal hypertension secondary to cirrhotic liver morphology with splenomegaly (*), enlarged splenorenal collateral vessels (*white arrows*) and enlarged left renal vein (*black arrow*). **b** Axial contrast-enhanced CT image shows dilatation of the pulmonary arteries with the main pulmonary artery measuring up to 3.4 cm in maximal diameter

Pulmonary arterial pressures greater than 25 mmHg at rest (as assessed by right-heart catheterisation) are diagnostic of pulmonary hypertension, with normal pressure being less than 20 mmHg [10, 11]. Rarely, pulmonary hypertension is idiopathic, commonly presenting in young women between 20 and 30 years of age. More often pulmonary hypertension is secondary to other pathology, with causes including heart failure (right and/or left), pulmonary parenchymal disease (including fibrosis and emphysema) and chronic pulmonary emboli. The current classification scheme of pulmonary hypertension (last updated in Nice, France) groups the causes of pulmonary hypertension into categories based on similar pathophysiology (Table 1) [12]. This framework is important as the aetiologies resulting in pulmonary hypertension often necessitate different therapeutic management. Imaging findings suggestive of pulmonary hypertension include an enlarged pulmonary trunk (> 29 mm), a pulmonary artery diameter to ascending aorta diameter of > 1, and a segmental artery-to-bronchus ratio > 1 in at least three of four lobes; additional imaging findings present may provide clues as to the underlying cause of the pulmonary hypertension (Figs. 5 and 6) [11, 13].

**Table 1:** Causes of pulmonary hypertension

Type of pulmonary hypertension	Comment
1. Pulmonary arterial hypertension	This category is broken down further into several subcategories. These categories include: idiopathic, heritable and drug/toxin induced. There is also a category for pulmonary arterial hypertension secondary to certain diseases, including congenital heart disease, connective tissue diseases, HIV infection, portal hypertension and schistosomiasis. Group 1′ includes pulmonary veno-occlusive disease (PVOD) and/or pulmonary capillary haemangiomatosis. Group 1″ is persistent pulmonary hypertension of the newborn.
2. Pulmonary hypertension secondary to left heart disease	This category may be caused due to left-heart disease of the valves, systolic dysfunction or diastolic dysfunction. The Nice classification added congenital/acquired left heart inflow/outflow tract obstruction along with congenital cardiomyopathies to this category.
3. Pulmonary hypertension with lung disease/hypoxaemia	This category may be caused by COPD, interstitial lung diseases, pulmonary diseases with a mixed restrictive and obstructive pattern, sleep-disordered breathing, alveolar hypoventilation disorders, chronic exposure to high altitude and developmental lung diseases.
4. Chronic thromboembolic pulmonary hypertension	This category arises in patients who have had chronic thromboembolic disease resulting in reorganisation of the pulmonary vasculature.
5. Pulmonary hypertension with unclear multifactorial mechanism	This is a broad category that includes pulmonary hypertension secondary to haematological disorders (including chronic haemolytic anaemia), systemic disorders (like sarcoidosis and lymphangioleiomyomatosis), metabolic disorders (like glycogen storage diseases or thyroid disorders) and other causes (including chronic renal failure and segmental pulmonary hypertension).

#### Pulmonary artery aneurysm/pseudoaneurysm

**Fig. 7:**
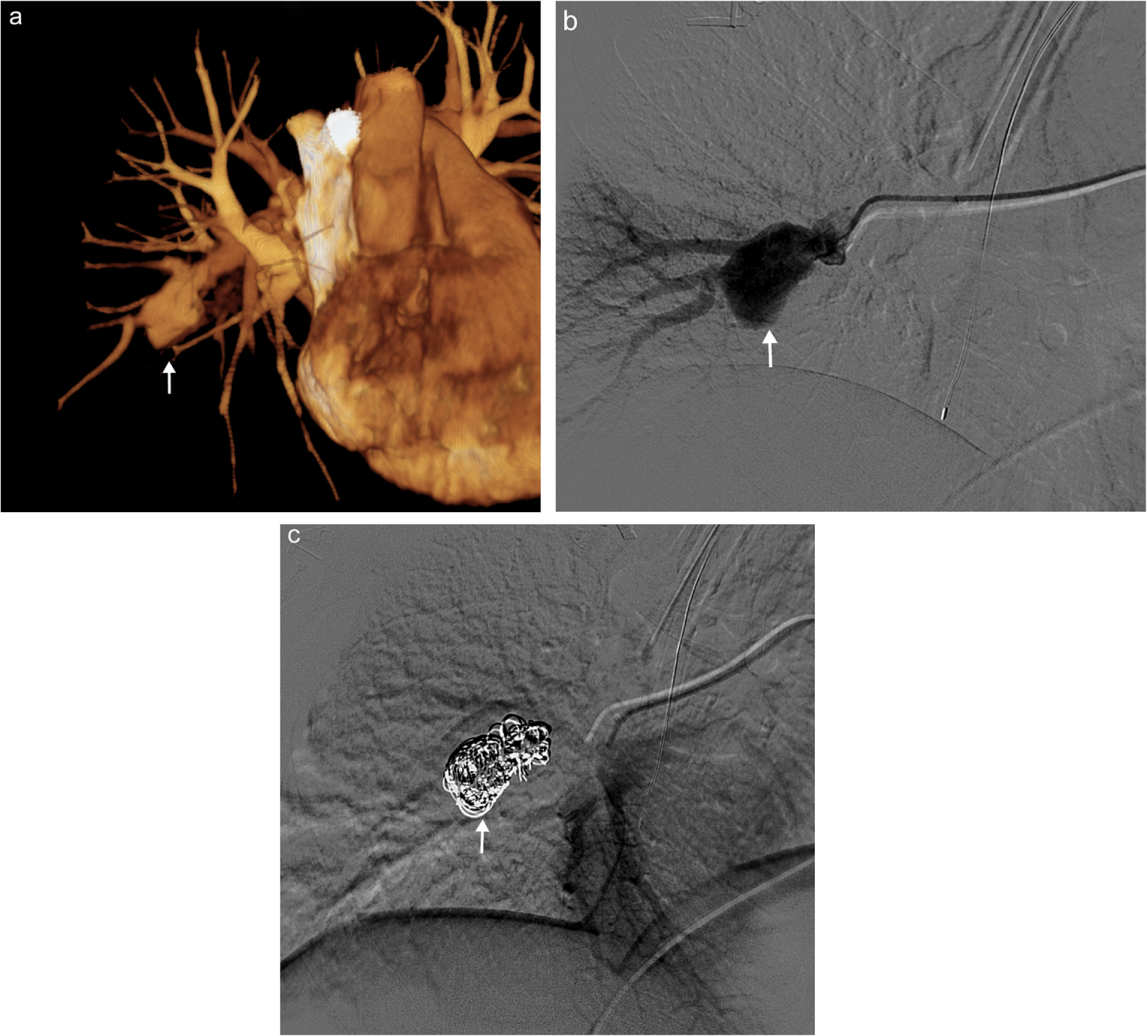
Pulmonary artery aneurysm in a patient with Behçet’s disease. **a** Three-dimensional reconstruction from a contrast-enhanced CT scan showing a right lower lobe pulmonary artery aneurysm in a patient with Behçet’s disease (*white arrow*). Digital subtraction fluoroscopic images obtained before (**b**) and after (**c**) coil embolisation of the right lower lobe pulmonary aneurysm (*white arrow*)

Aneurysms/pseudoaneurysms of the pulmonary artery may arise secondary to pulmonary hypertension, collagen/vascular conditions (e.g. Marfan syndrome, Takayasu arteritis and Behçet’s disease), iatrogenic catheter misplacement, trauma and infection. They may occur in isolation or may also be associated with other congenital cardiac disease. The normal pulmonary artery should gently taper in calibre as the vessel branches and extends peripherally. Imaging will reveal a focal dilated segment of the pulmonary artery, often saccular in appearance, with the phase of contrast following the pulmonary artery enhancement (Fig. 7) [1].

#### Pulmonary artery narrowing/obstruction

**Fig. 8:**
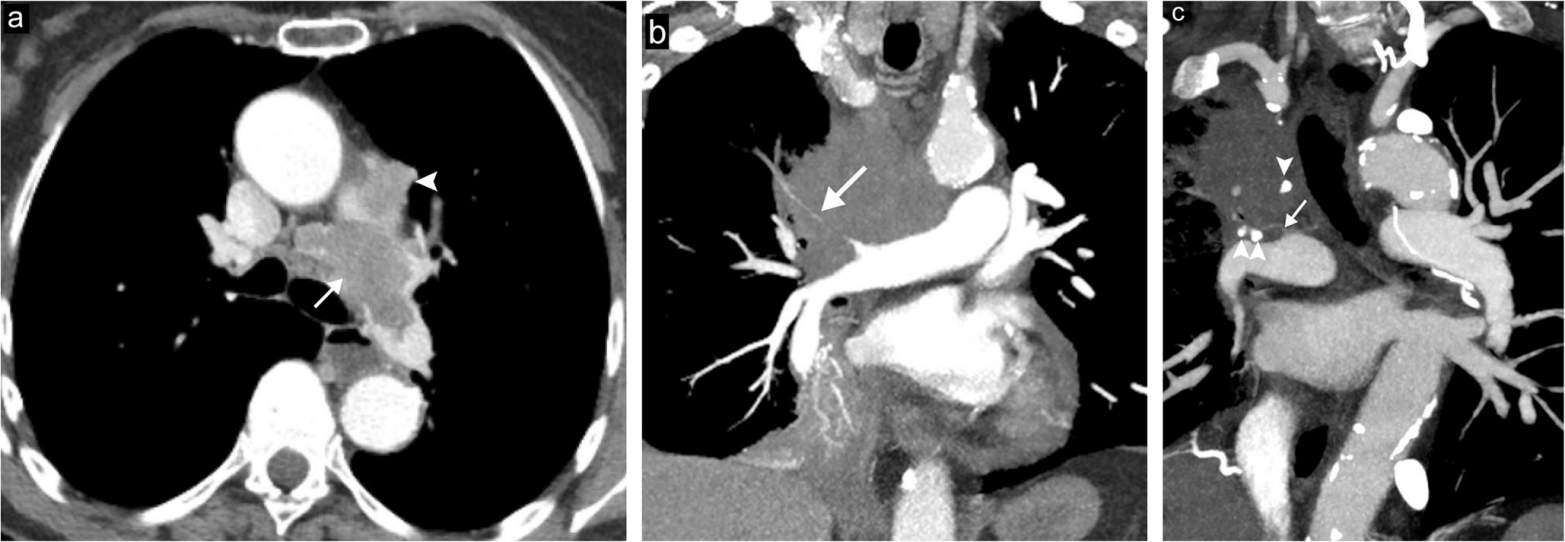
Pulmonary artery narrowing/obstruction. **a** Axial contrast-enhanced CT image shows a large, irregularly shaped filling defect within the left pulmonary artery (*arrow*) with extension into the mediastinal fat (*arrowhead*) causing intrinsic narrowing/obstruction of the pulmonary artery in a patient with biopsy proven angiosarcoma. **b** Coronal CT image demonstrating severe narrowing of the right upper lobe pulmonary artery (*arrow*) secondary to extrinsic mass effect from a right upper lobe primary lung malignancy. **c** Coronal CT image demonstrating complete occlusion of the right upper lobe pulmonary artery (*arrow*) secondary to extrinsic compression from biopsy proven fibrosing mediastinitis. Note the coarse calcifications present within the region of fibrosis (*arrowheads*)

Obstruction of the pulmonary artery can occur secondary to malignant processes including both primary sarcoma of the pulmonary artery and other pulmonary neoplasms, inflammatory conditions including mediastinal fibrosis and Takayasu arteritis, or pulmonary embolism both acute and chronic. Primary sarcoma of the pulmonary arteries is a rare condition and is often confused for acute pulmonary embolism. CT imaging will show an enlarged artery with a filling defect that may encompass the entire lumen (Fig. 8a); findings that should raise the suspicious for sarcoma over bland thrombus include entire involvement of the proximal or main pulmonary artery, enlargement of the involved pulmonary arteries, and extension of the lesion into the surrounding tissues [14]. Occasionally, small enhancing vessels can also be seen within the tumour itself. Malignant pulmonary neoplasms can cause external compression or directly invade the pulmonary artery resulting in obstruction (Fig. 8b) [1]. Fibrosing mediastinitis is due to the pathological proliferation of fibrous tissue in the mediastinum and may also cause obstruction or narrowing when the pulmonary arteries are involved (Fig. 8c) [15]. While more commonly affecting the aorta, Takayasu arteritis also can affect the pulmonary arteries with CT angiography typically revealing circumferential wall thickening and enhancement [16].

**Fig. 9:**
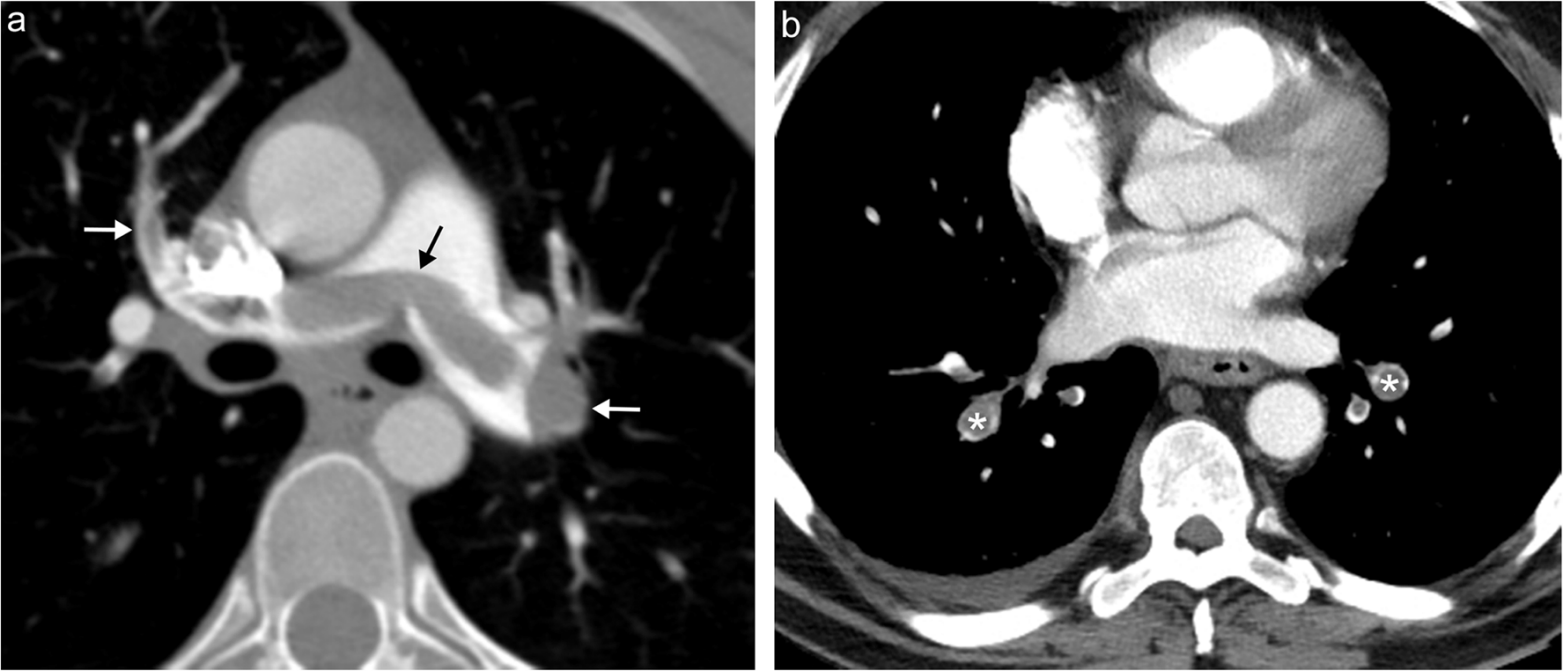
Two patients with acute pulmonary emboli. **a** Axial contrast-enhanced CT image shows a large saddle embolus of the main pulmonary artery (*black arrow*) as well as lobar/segmental involvement of the right and left pulmonary arteries (*white arrows*). **b** Axial contrast-enhanced CT image of another patient with bilateral acute pulmonary emboli demonstrate the central focal filling defect within bilateral segmental pulmonary arteries which is rimmed by contrast (*)

**Fig. 10:**
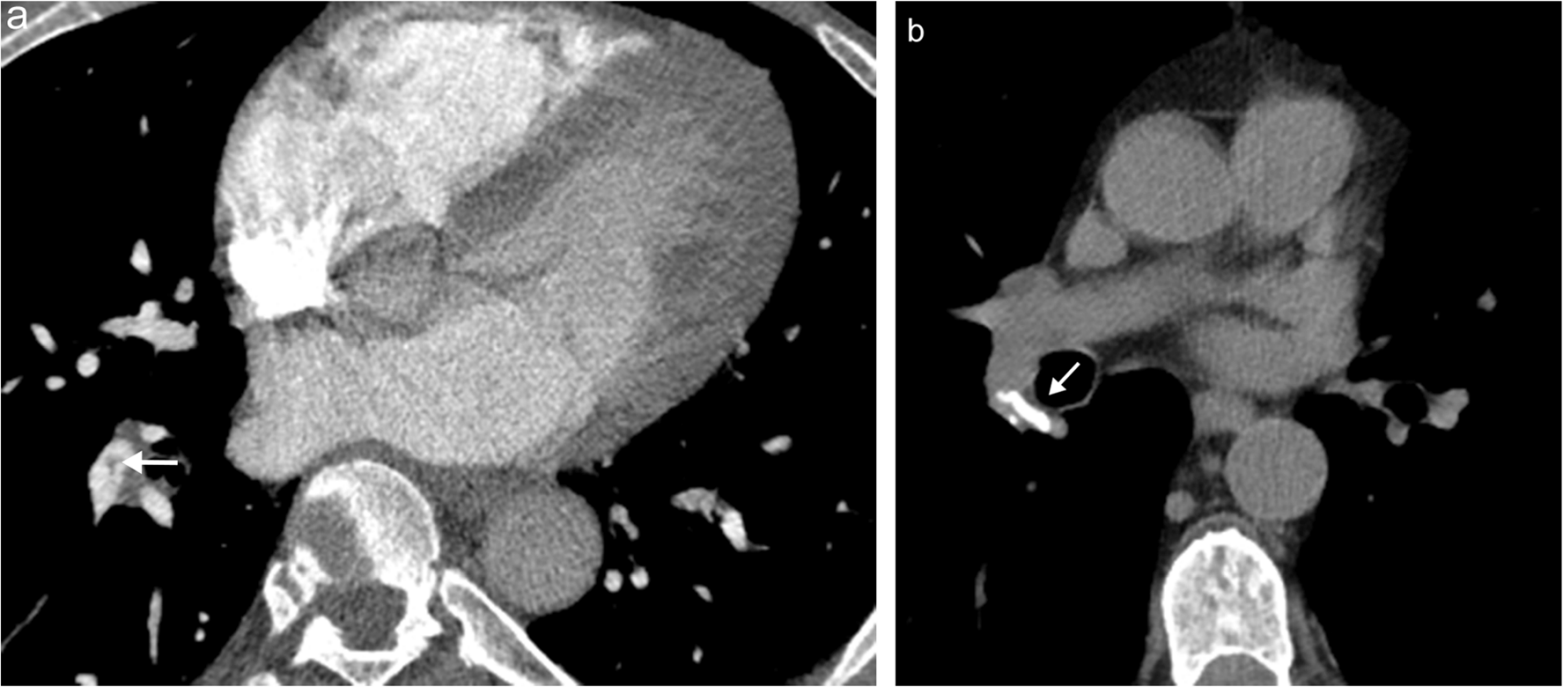
Two patients with chronic pulmonary emboli. **a** Axial contrast-enhanced CT image shows a web-like filling defect within a segmental right lower lobe pulmonary artery consistent with chronic pulmonary embolus (*white arrow*). **b** Axial non-contrast CT image of another patient demonstrates linear calcification within the right interlobar pulmonary artery consistent with chronic pulmonary embolus (*white arrow*)

Pulmonary embolism is a very common source of pulmonary artery obstruction. Acute pulmonary embolism is best evaluated with CT pulmonary angiography which demonstrates a central focal filling defect within the pulmonary artery, often rimmed by contrast, typically forming an acute angle within the vessel. Enlargement of the affected branch may also be seen (Fig. 9) [17, 18]. In some patients (especially those with repeat episodes or particularly large emboli) chronic pulmonary thromboembolism can develop with the prior embolus becoming incorporated into the luminal wall rendering anticoagulation less effective. This leads to stenosis of the pulmonary artery and may cause serious pulmonary hypertension. CT findings in chronic pulmonary thromboembolism include eccentric thrombus resulting in partial or complete obstruction, often making an obtuse angle with the vessel wall, post stenotic dilatation, partial calcification of the thrombus, and web-like bands within the pulmonary arteries. Indirect signs include mosaic attenuation of the pulmonary parenchyma due to perfusion defects and APC formation (Fig. 10) [19].

## Pulmonary vein abnormalities

### Congenital disorders

#### Congenital variations in number

**Fig. 11:**
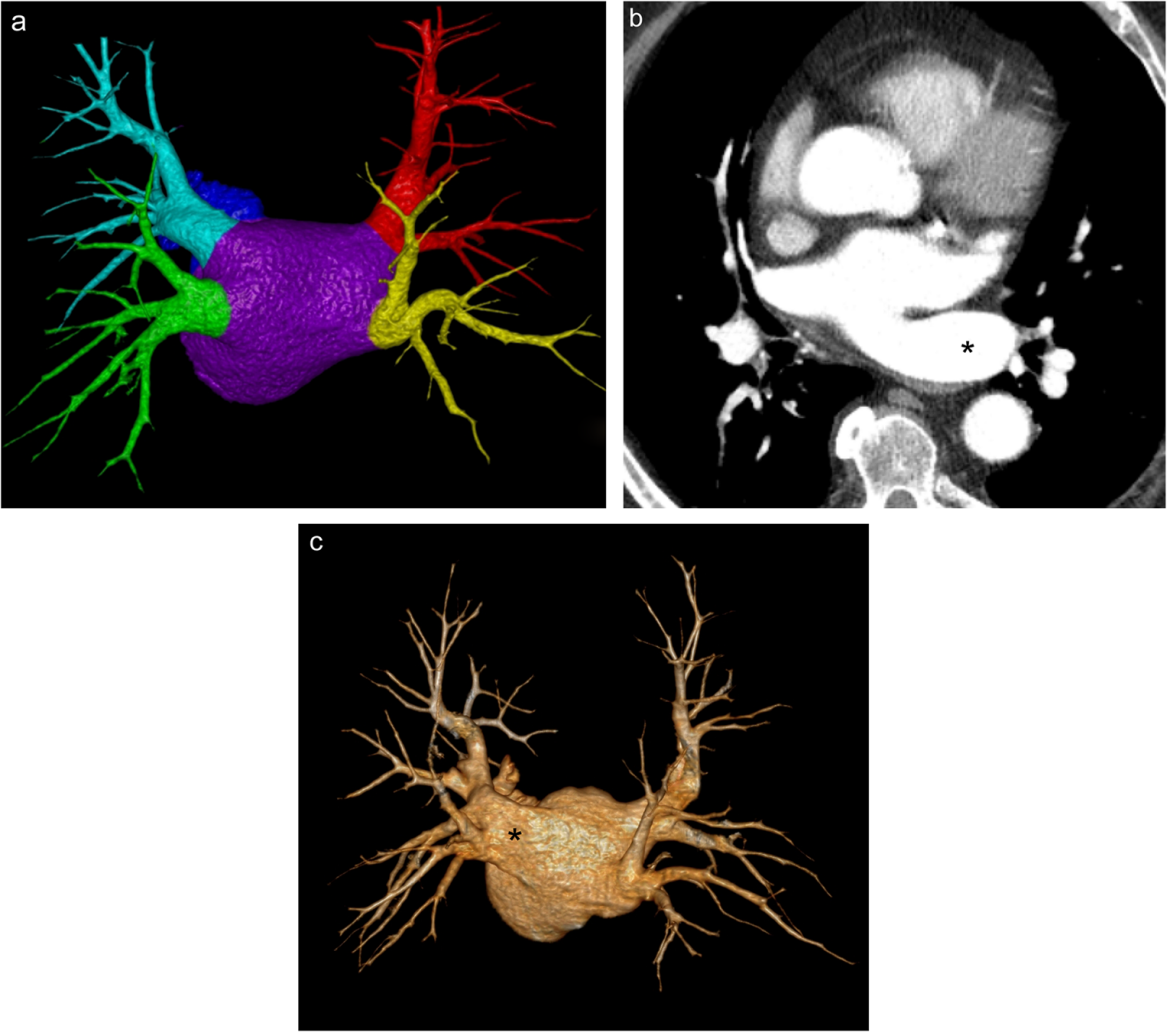
Pulmonary venous variant. **a** Three-dimensional reconstruction of a contrast-enhanced CT shows normal pulmonary venous anatomy with two right and two left pulmonary veins draining into the left atrium. **b** Axial CT image showing the most common variant of pulmonary venous anatomy, a fusion of the left pulmonary veins prior to entry in the left atrium (*). **c** Three-dimensional reconstruction shows a fusion of the left superior and inferior pulmonary veins with a shared ostium (*)

Anatomically, the normal configuration of the pulmonary venous territory involves four distinct pulmonary veins draining into four separate ostia on the left atrium (two right and two left); however, anatomic variants are common. A particularly common variant involves a common left pulmonary vein, where the superior and inferior left pulmonary veins fuse before draining into the left atrium (Fig. 11) [20]. Please note that the merging of the pulmonary veins should be distinguished from an anomalous unilateral single pulmonary vein, a condition where there is only a single pulmonary vein without merger (more common on the left side) [21]. A second common variant is a separate right middle lobe pulmonary vein draining independently from the superior and inferior right pulmonary veins. Other anatomical variants have been described such as the presence of more than two pulmonary veins on the same side [22].

#### Anomalous pulmonary venous return

**Fig. 12:**
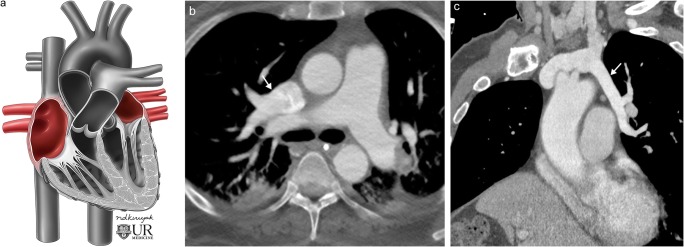
Partial anomalous pulmonary venous return. **a** Conceptual illustration of partial anomalous pulmonary venous return. **b** Axial contrast-enhanced CT of an adult patient with partial anomalous venous return of the right upper lobe (*white arrow*) with blood flow returning to the superior vena cava. **c** Double oblique contrast-enhanced CT of an adult patient with partial anomalous venous return of the left upper lobe (*white arrow*) with blood flow returning to the left brachiocephalic vein

**Fig. 13:**
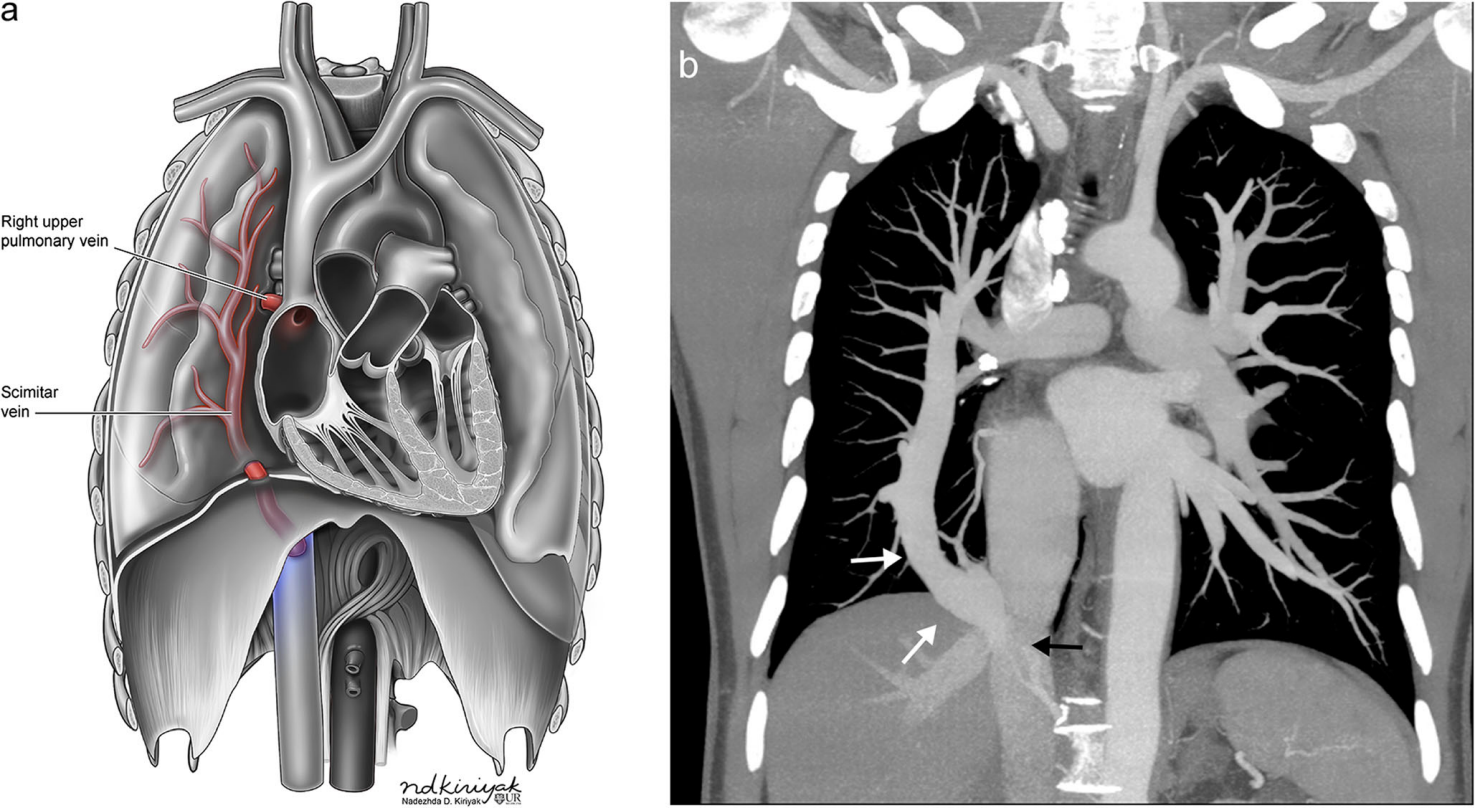
Scimitar syndrome. **a** Conceptual illustration of scimitar syndrome. **b** Coronal contrast-enhanced CT of an adult patient with scimitar syndrome showing the entire right lung pulmonary venous return (*white arrows*) draining into the inferior vena cava (*black arrow*)

Congenital pulmonary venous drainage anomalies refer to conditions in which pulmonary vein flow does not return to the left atrium but rather drains into the systemic system producing a left-to-right shunt. This pathology exists on a wide spectrum with tremendous variability. Total anomalous pulmonary venous return (TAPVR) occurs when all pulmonary venous return drains into the systemic system. TAPVR is classified into supracardiac, cardiac, infracardiac or mixed types depending on the location of the drainage. This is a condition incompatible with life and requires an accompanying right-to-left shunt for survival, along with immediate corrective surgery [23, 24]. TAPVR has several associations including heterotaxy syndrome and other congenital heart defects [24]. Partial anomalous pulmonary venous return (PAPVR) refers to conditions in which only some of the pulmonary veins drain aberrantly (Fig. 12) [2, 24, 25]. The severity of PAPVR depends on the degree of anomalous return, and these patients are often asymptomatic. A special subtype of PAPVR occurs when there is aberrant, typically right sided, pulmonary venous drainage into the inferior vena cava (or other veins below the diaphragm) with associated hypoplasia of the right lung. This condition is known as hypogenetic lung syndrome due to the associated small right lung or scimitar syndrome due to the scimitar sword-like appearance of the aberrant venous return extending below the diaphragm on frontal radiograph. The degree of PAPVR is a function of the severity of the disease process, with larger defects (such as aberrant entire return on one side) often identified in infancy and smaller defects (such as aberrant lobar or segmental return) often remaining asymptomatic, identified incidentally in adulthood (Fig. 13) [23, 26]. Treatment of all these conditions range from observation in mild cases to emergent surgery in more severe manifestations.

#### Pulmonary vein varix

**Fig. 14:**
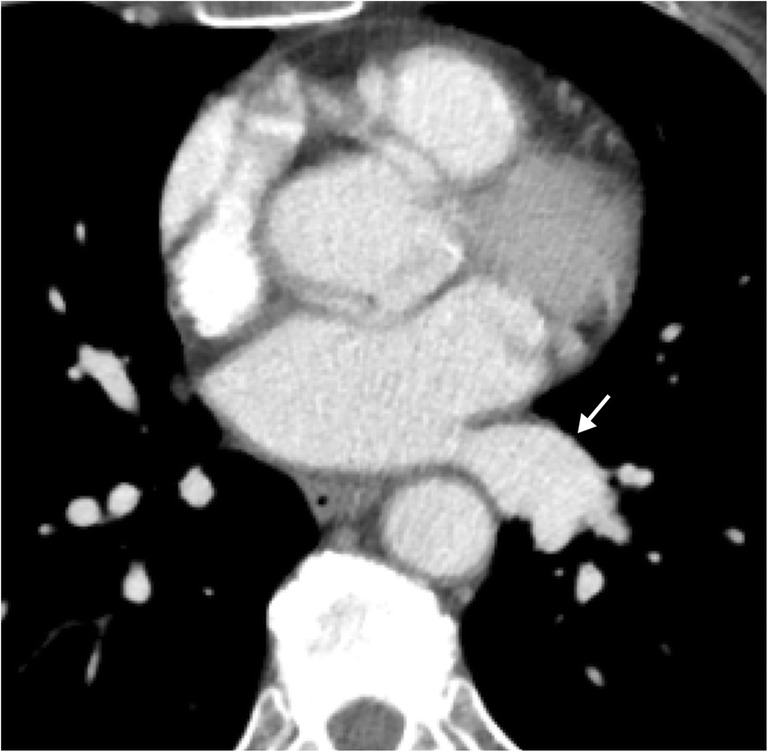
Pulmonary vein varix. Axial contrast-enhanced CT image demonstrated a pulmonary vein varix (*white arrow*); note its proximity to the insertion in the left atrium. Varices can be saccular, tortuous, or confluent, with confluent being the most common

An isolated segment of dilated pulmonary vein is known as a varix or pulmonary venous aneurysm [27]. This dilatation is readily apparent on CT imaging and occurs most frequently near the ostium (Fig. 14). On chest X-ray, the varix may assume the appearance of a pulmonary nodule or hilar adenopathy [28]. Although we have grouped the pulmonary vein varix with congenital conditions, it can also be acquired secondary to other cardiopulmonary disease such as chronic pulmonary hypertension [2]. Pulmonary vein varices are typically asymptomatic; however, rupture and subsequent haemorrhage are known complications requiring treatment that may involve surgery or coiling [28].

#### Pulmonary vein congenital atresia/stenosis

Failure of the pulmonary vein to properly develop may result in atresia or stenosis. Patients will classically present with recurrent pneumonia and/or haemoptysis in the first few years of life, and there is an association with other congenital cardiac anomalies [29]. CT imaging may show ipsilateral mediastinal deviation towards the atretic pulmonary vein, a small ipsilateral hemithorax and a small ipsilateral pulmonary artery [2].

### Acquired disorders

#### Pulmonary vein stenosis/obstruction

**Fig. 15:**
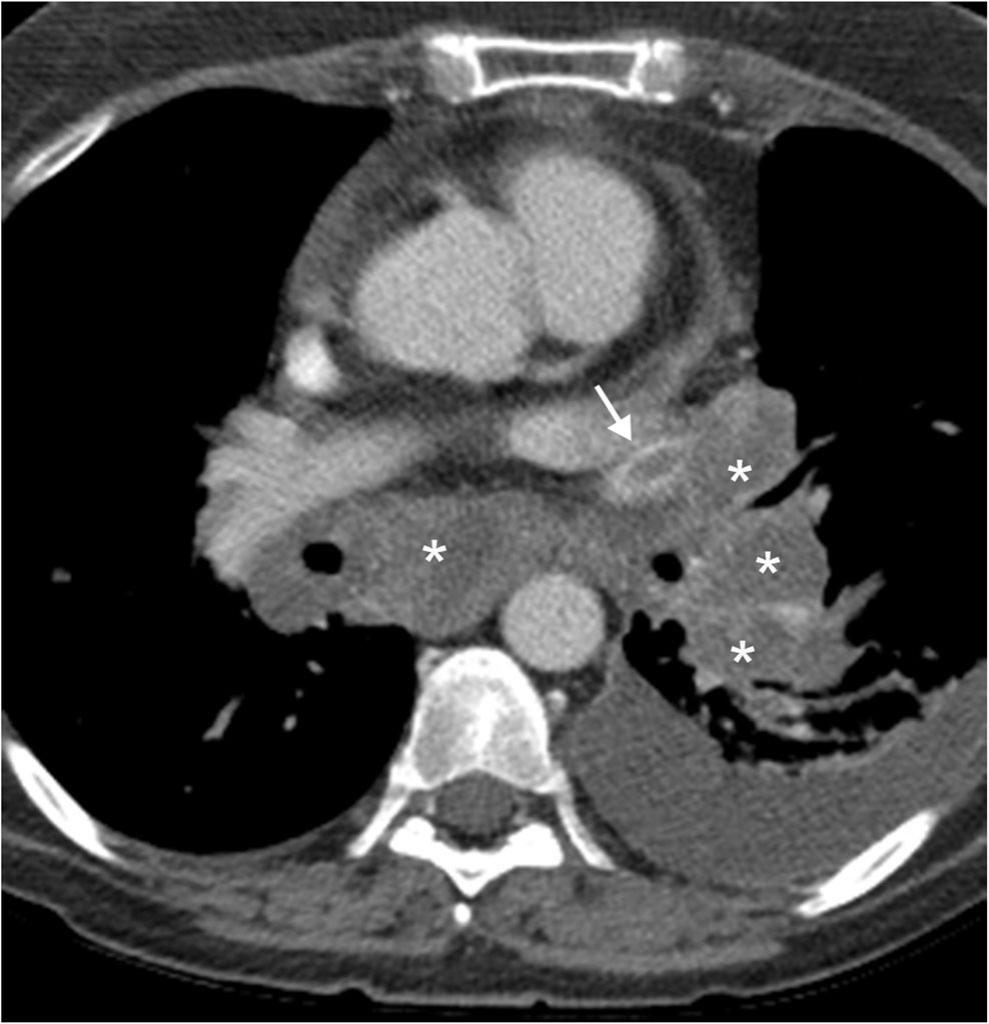
Pulmonary venous thrombus. Axial contrast-enhanced CT image in a patient with non-small cell lung cancer demonstrating a filling defect within the left superior pulmonary vein (*white arrow*). Note extensive lymphadenopathy within the left hila and mediastinum (*) from metastatic involvement

There are many conditions which may lead to the obstruction of pulmonary venous flow, including both intrinsic and extrinsic processes. The majority of cases relate to neoplastic processes or complications of radiofrequency ablation; other causes include surgical complications, fibrosing mediastinitis, tuberculosis and sarcoidosis [2, 15, 30–32]. As with pulmonary arteries, neoplastic processes can result in either mechanical extrinsic compression or direct invasion of the pulmonary venous vasculature causing obstruction. While this typically results in stenosis or occlusion of the vessel, it can also lead to pulmonary vein thrombosis, with bland thrombus developing secondary to disrupted flow, hypercoagulable state or tumour thrombus from direct extension of tumour or metastasis. Other causes of stenosis/obstruction, such as pulmonary surgery or radiofrequency ablation, can also cause bland thrombus [33, 34]. Clinical presentation of thrombus or stenosis/obstruction in pulmonary veins is non-specific. Obstruction of the pulmonary veins can be detected on CT imaging with contrast; however, this is a commonly overlooked finding as the pulmonary veins are not typically analysed in detail during a general search pattern for CT chest imaging (Fig. 15) [35].

#### Pulmonary vein collateral pathways

**Fig. 16:**
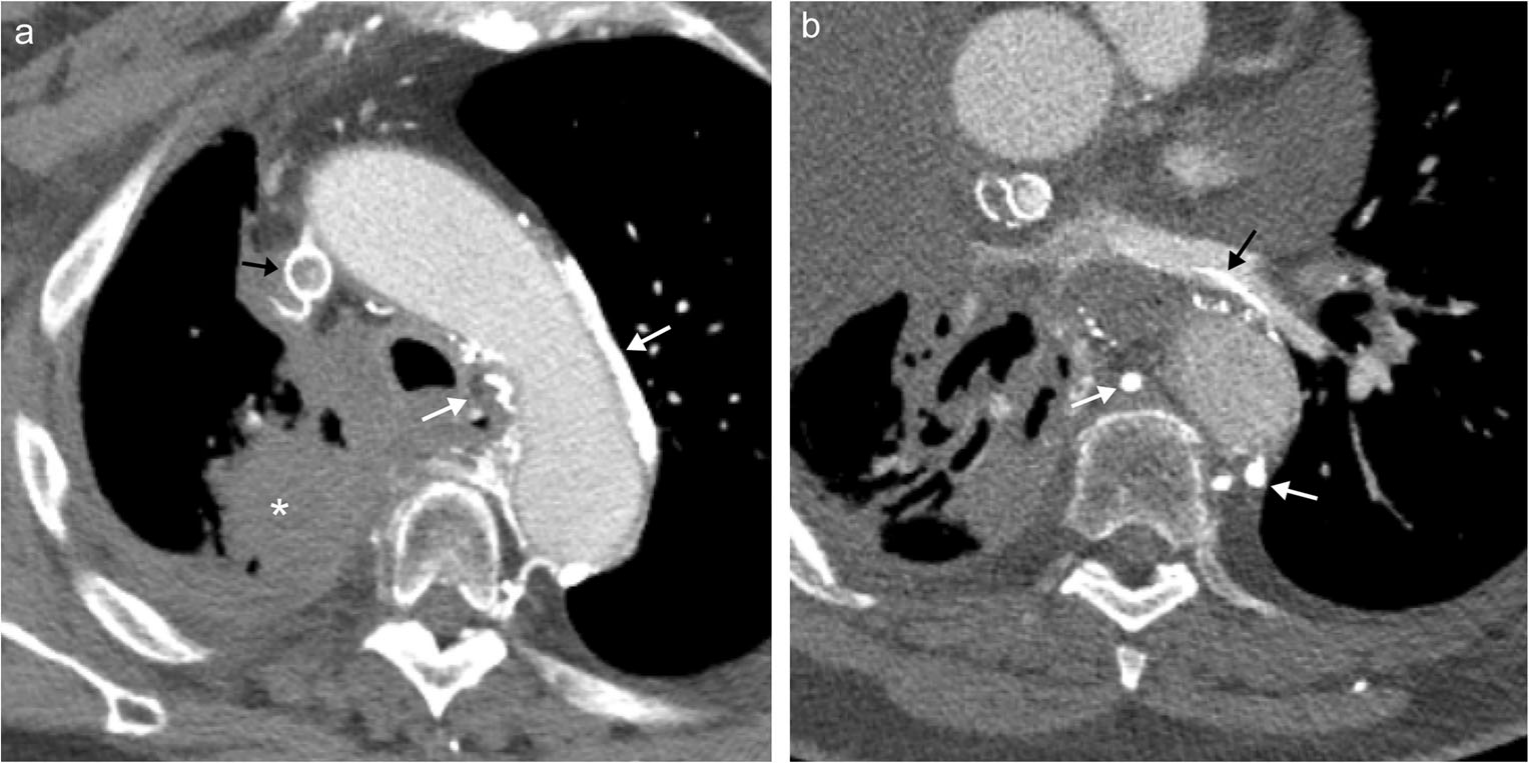
Pulmonary venous collaterals. **a** Axial contrast-enhanced CT image demonstrates upper right lobe squamous cell carcinoma (*) with metastatic disease obstructing the superior vena cava and stent (*black arrow*). Note extensive collateral formation (*white arrows*). **b** Axial CT image showing high-density contrast flow into the pulmonary venous system (*black arrow*) secondary to collateral formation (*white arrows*) with a right-to-left shunt in the setting of superior vena cava obstruction

It is important to note that the pulmonary veins can be involved in a variety of collateral pathways, both congenital and acquired [2]. In cases of superior vena cava obstruction, collateral flow to the pulmonary veins can form, resulting in a right-to-left shunt (Fig. 16) [36, 37].

#### Pulmonary venous hypertension

Pulmonary venous hypertension (defined as a pressure of 15 mmHg or higher) occurs most often in the background of left ventricular failure or other cardiac causes. A rare idiopathic cause of pulmonary venous hypertension is known as pulmonary veno-occlusive disease, which develops secondary to the occlusion or constriction of the pulmonary veins and venules, with the majority of patients affected presenting under the age of 50 years old [2, 38]. Patients with pulmonary venous hypertension show evidence of fluid overload on imaging, including pulmonary oedema and pleural effusions, mosaic attenuation of the pulmonary parenchyma and enlargement of the central pulmonary arteries [13].

#### Pulmonary vein calcifications

Pulmonary vein calcifications are typically associated with mitral valve disease and chronic renal failure; however, they may also be seen in patients with atrial fibrillation [2, 39]. In addition to pulmonary vein calcifications, imaging often shows a dilated left atrium, which may also be calcified, described as a “mould-like” pattern [40].

## Other pulmonary vascular abnormalities

### Pulmonary arteriovenous malformations

**Fig. 17:**
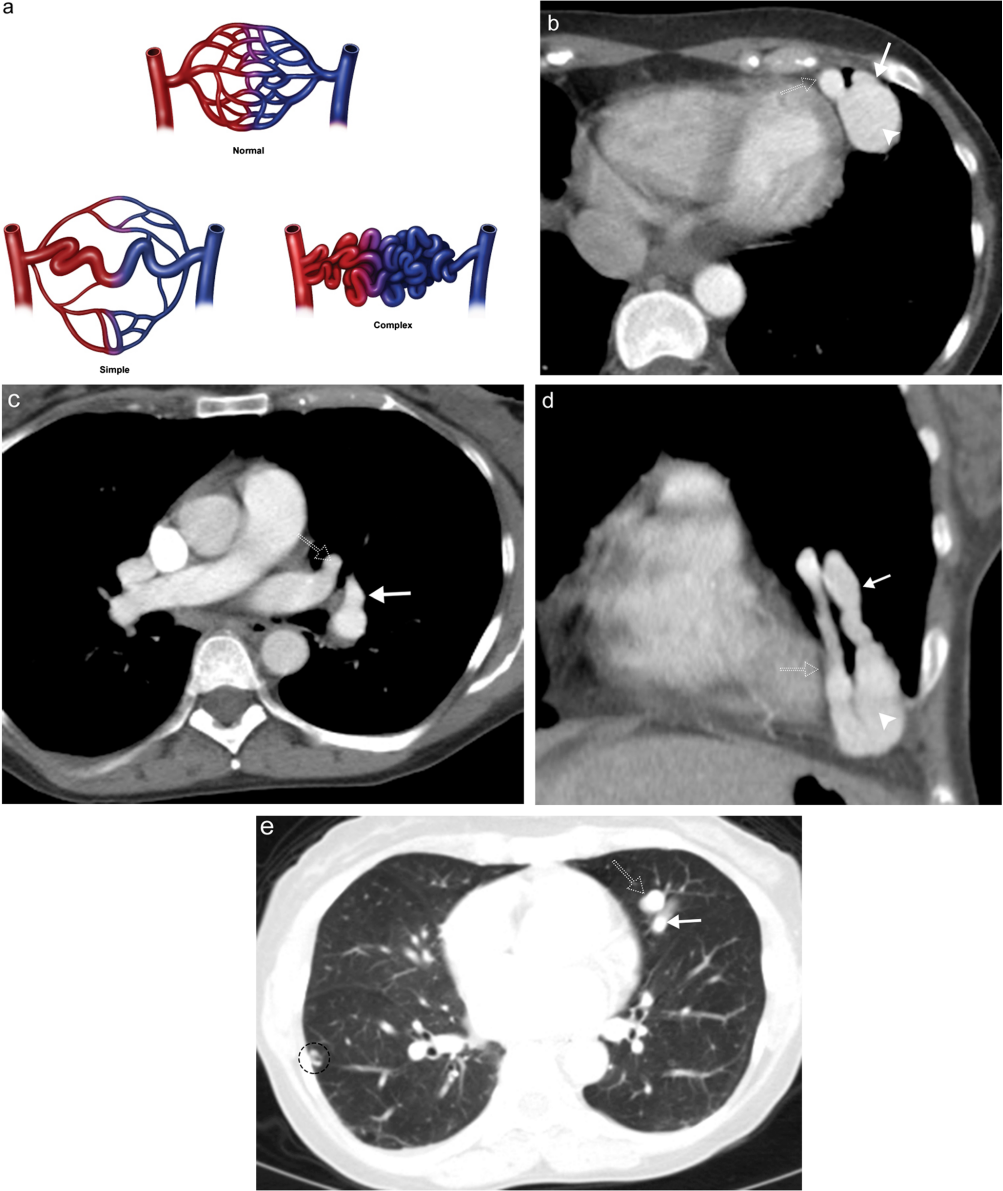
Pulmonary arteriovenous malformation. **a** Conceptual illustration of simple versus complex arteriovenous malformation. Axial (**b, c**) and coronal (**d**) contrast-enhanced CT images demonstrate a large arteriovenous malformation of the left upper lobe (*arrowhead*) with a clearly identifiable feeding pulmonary artery (*solid arrow*) and draining pulmonary vein (*dotted arrow*). **e** Axial contrast-enhanced CT images of the same patient in lung windows reinforces the importance of fully evaluating the entire lungs as additional smaller AVMs (*circle*) were also identified in this patient leading to the diagnosis of hereditary haemorrhagic telangiectasia

Pulmonary arteriovenous malformations (AVMs) are pathological connections between the pulmonary artery and the pulmonary vein without an intermediary capillary network resulting in right-to-left shunt [41]. Pulmonary AVMs are typically congenital, thought to be present at birth, but typically only become clinically relevant in adulthood. Hereditary haemorrhagic telangiectasia should be considered if more than one AVM is identified, as this condition is associated with multi-organ AVMs involving the central nervous system, liver, skin, mucous membranes and gastrointestinal tract. Pulmonary AVMs can also be acquired, resulting from trauma, surgery for congenital heart disease, tuberculosis and schistosomiasis [42]. Simple AVMs involve a single segmental feeding artery, while complex malformations involve multiple feeding arteries; diffuse arteriovenous malformations may manifest as hundreds of lesions, which can be both simple and complex (Fig. 17) [43]. Lesions are best imaged by CT, which will show a well-circumscribed non-calcified nodule with associated enlarged feeding artery(s) and draining vein(s), often with lower lobe predominance (Fig. 17) [28, 44]. Occasionally, calcified phleboliths may be present. While contrast is not needed for this evaluation, early-phase/pulmonary arterial phase imaging typically shows enhancement of the feeding artery, aneurysmal nodule and draining vein. Clinical presentation depends on the size of the malformation, ranging from asymptomatic to dyspnoea, hypoxaemia, pulmonary hypertension and paradoxical emboli [2]. Lesions greater than 2 cm require treatment with percutaneous transcatheter embolisation or surgical intervention.

### Pulmonary sequestration

**Fig. 18:**
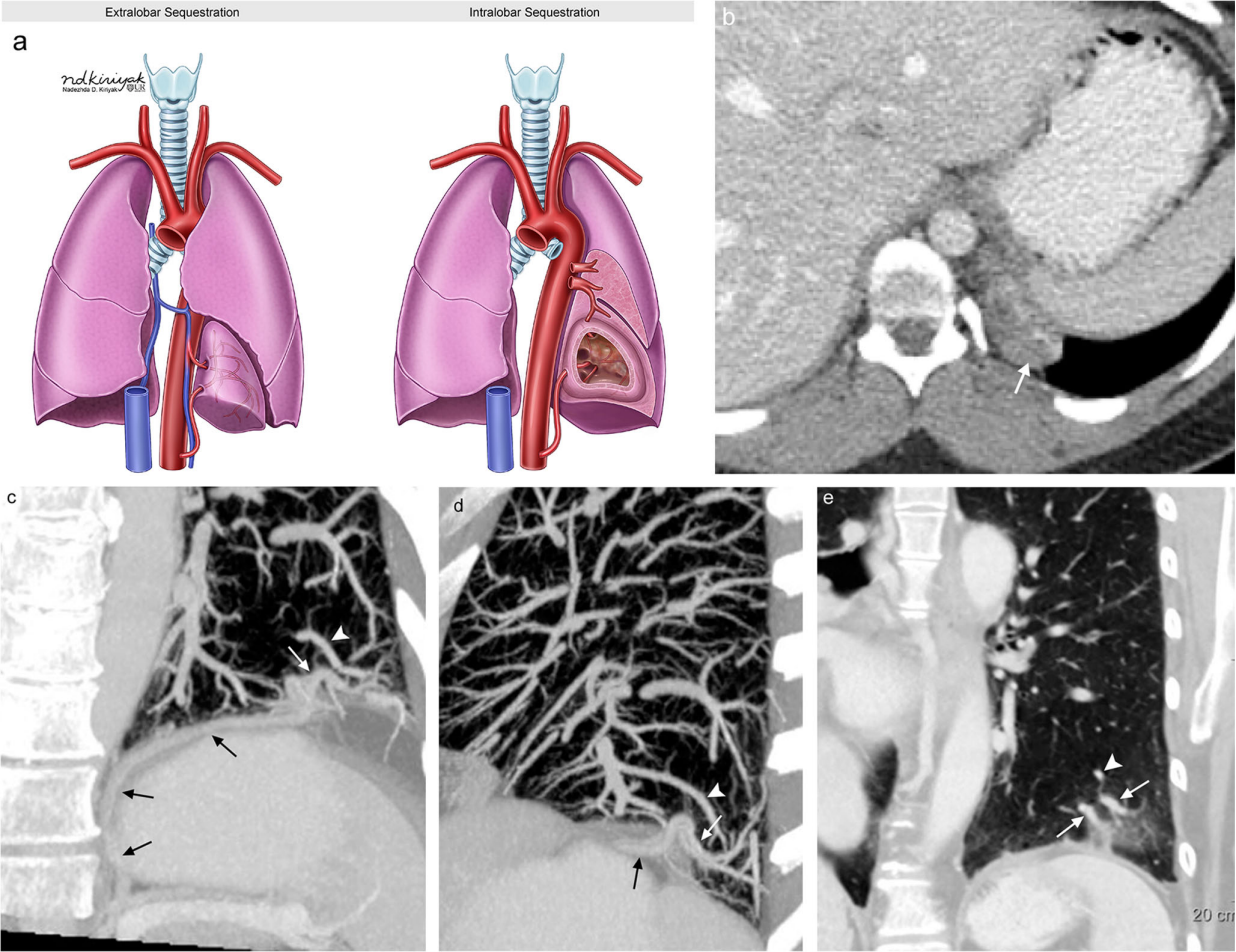
Pulmonary sequestration. **a** Conceptual illustration of extralobar and intralobar sequestration. Note that extralobar sequestration involves venous drainage into the azygos system forming a left-to-right shunt while intralobar sequestration involves pulmonary venous drainage forming a left-to-left shunt. **b** Axial CT demonstrating an asymptomatic oval lesion (*white arrow*) in the left lower lobe consistent with extralobar sequestration in a 23-year-old woman. Blood supply is derived from the abdominal aorta with venous drainage occurring via the azygos system. Double oblique CT MIP images (**c, d**) and coronal CT MPR image (**e**) demonstrating an intralobar sequestration located within the pleura of the left lower lobe. Note the aberrant arterial supply to the lateral left lower lobe below the level of the diaphragm (*black arrows*) which extends through the diaphragm into the tissues of the left lower lobe (*white arrows*). Venous drainage of this intralobar sequestration is via the pulmonary venous system (*arrowheads*)

Pulmonary sequestration is a rare developmental abnormality characterised by non-functional, aberrant lung tissue without a connection to the tracheobronchial tree, yet still receiving systemic arterial flow (typically from the aorta) [45]. There are two types of pulmonary sequestration: intralobar and extralobar sequestration (Fig. 18). While these two pathological processes both involve aberrant lung tissue (almost exclusively in the lower lobe) with systemic arterial blood supply, they differ in respect to their relationship with the pleura, venous drainage and clinical presentation [28]. Intralobar sequestration (making up 75% of pulmonary sequestration cases) stereotypically presents in childhood with recurrent pulmonary infections. This portion of the lung is closely associated to the adjacent normal lung, without a separate pleural surface, and the venous drainage is typically via the pulmonary venous system, creating a left-to-left shunt. Please note that venous drainage via the vena cava, azygous veins or direct connection to the right atrium may also occur creating a left-to-right shunt. In contrast, extralobar sequestration (also known as accessory lung) is covered by its own pleura with venous drainage typically involving the azygos and hemiazygos system to the right atrium creating a left-to-right shunt. Extralobar sequestration is typically diagnosed during infancy with difficulty feeding or episodes of cyanosis; it is also associated with several other developmental abnormalities including scimitar syndrome and congenital heart disease [46, 47].

## Bronchial artery abnormalities

**Fig. 19:**
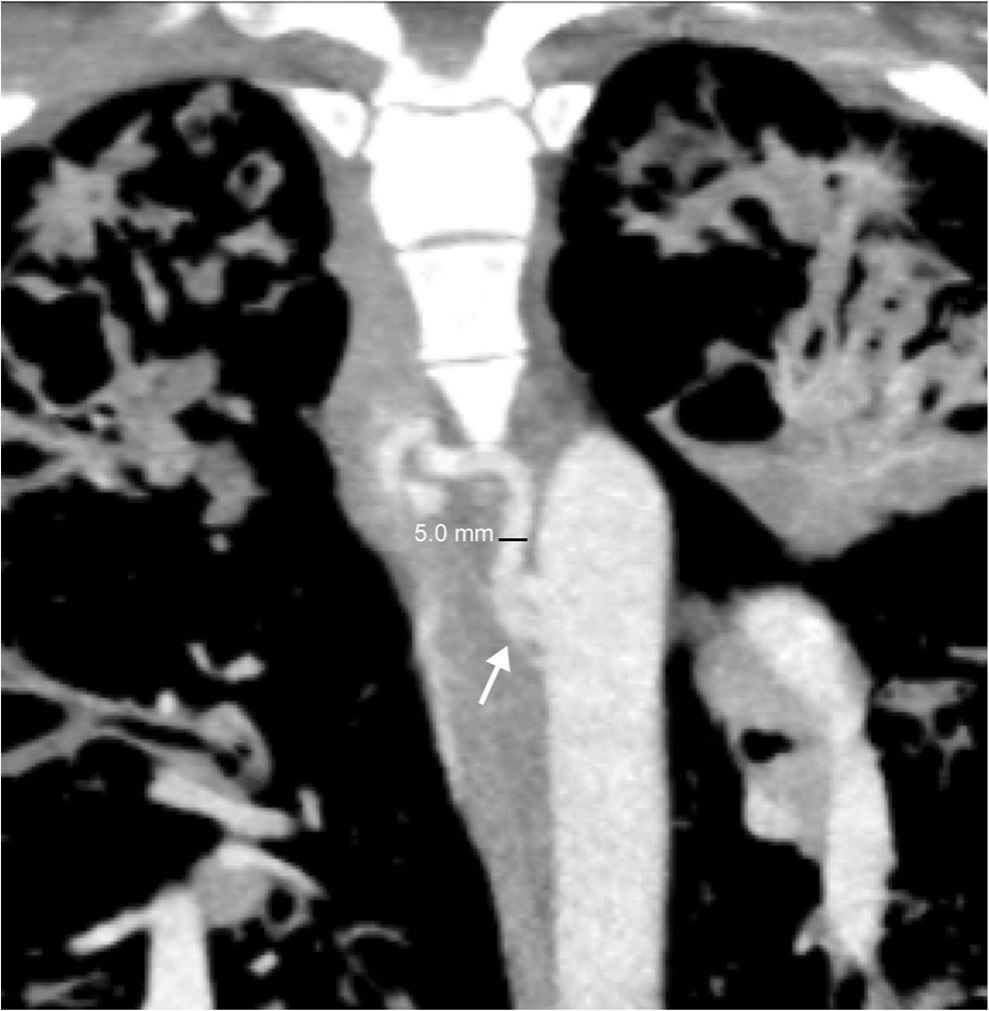
Bronchial artery anatomy. Coronal CT of the chest shows a dilated right bronchial artery (5 mm) arising from the intercostal bronchial artery trunk (ICBAT) (*white arrow*) of the postero-lateral thoracic aorta at the level of T6. Please note that the normal bronchial artery is less than 2 mm in diameter. While ICBAT origin is the most typical of the right bronchial artery, the left bronchial arteries usually arise directly from the aorta

**Fig. 20:**
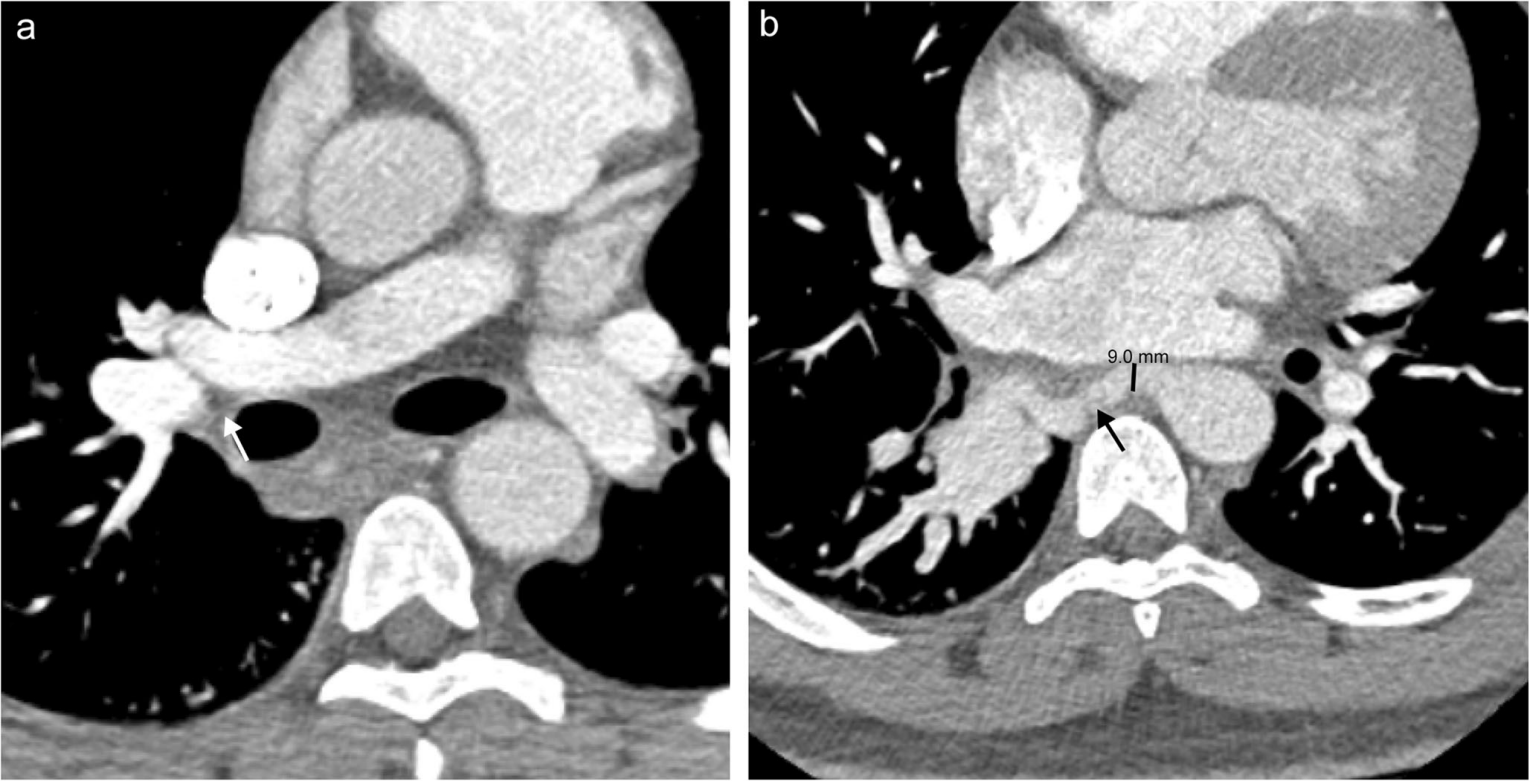
Bronchial artery dilatation. **a** Axial CT image showing congenital absence of the right lower lobe pulmonary artery (*white arrow*). **b** Axial CT image showing 9.0 mm dilated bronchial artery supplying the right lower lobe (*black arrow*)

Bronchial arteries typically branch off the descending thoracic aorta but may also originate from the subclavian arteries. There are classically two left and one right bronchial artery, which measure less than 2 mm in size. Although there is much variation in origin, it is important to note that the right bronchial artery typically shares a common origin with an intercostal artery (termed an intercostal-bronchial artery trunk or ICBAT) (Fig. 19). The bronchial vascular system is responsible for supplying the nutrition to the pulmonary parenchyma and is usually not involved in alveolar gas exchange. However, in response to insufficient blood flow to the lungs, the bronchial vessels will undergo hypertrophy and often form collateral pathways to the pulmonary arterial system via the anatomic microscopic vessels which connect the pulmonary and bronchial systems in healthy individuals (Fig. 20) [5, 48, 49]. Although typically making up ~ 1% of the cardiac output, dilated bronchial arteries can utilise up to 20% of the entire cardiac output in disease states [50].

Dilated bronchial arteries (> 2 mm) are often a sign of serious pathology and should prompt immediate investigation for an underlying cause (Fig. 20) [51]. Several causes of bronchial artery dilation have been discussed in the previous sections, including congenital disorders of the heart and pulmonary artery, pulmonary artery obstruction and inflammatory conditions of the lungs [5]. Of note, patients with idiopathic pulmonary hypertension do not typically have dilatation of the bronchial vasculature, providing a helpful tool in differential diagnosis [52].

**Fig. 21:**
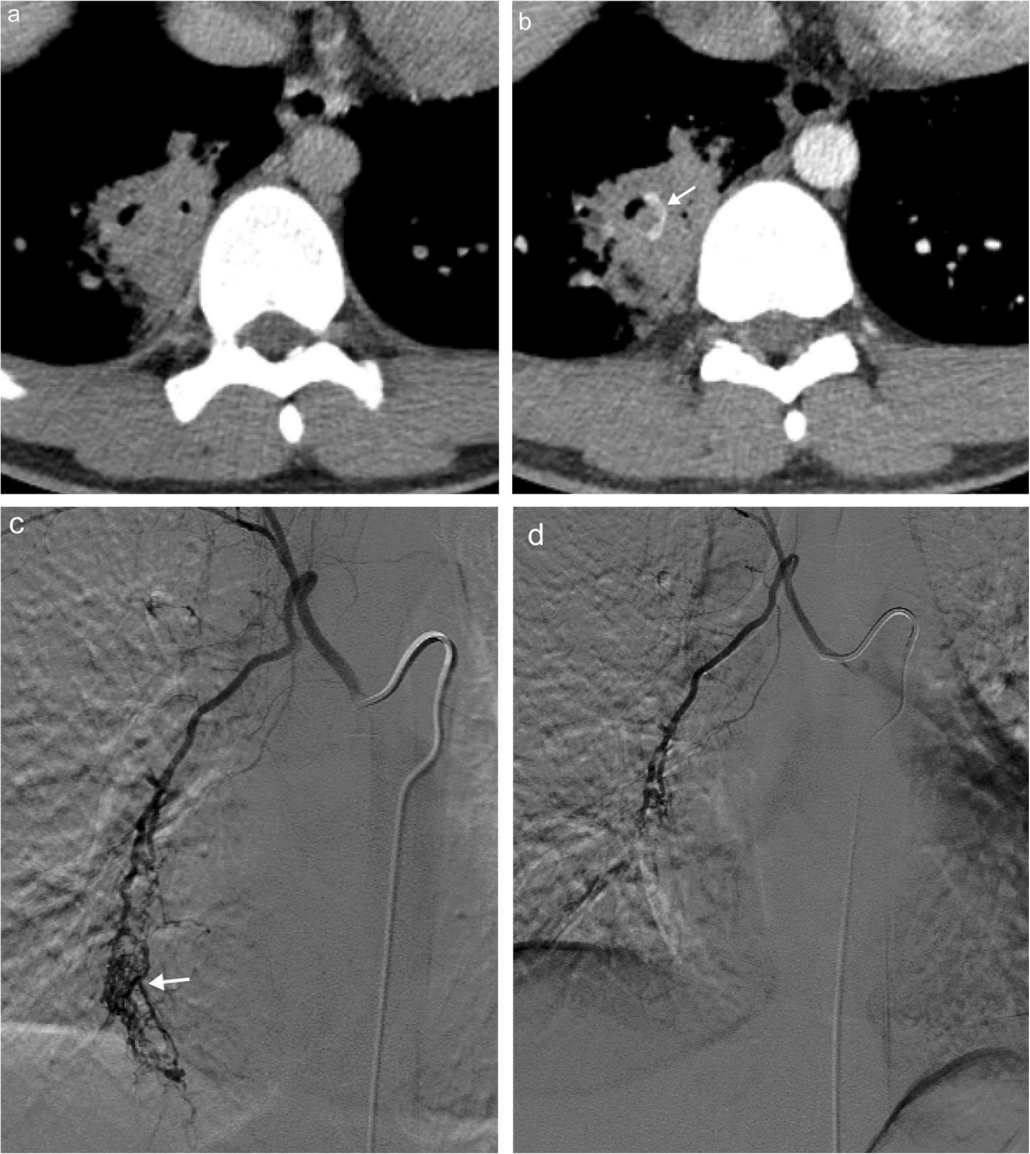
Bronchial artery bleeding. Pre- (**a**) and post-contrast (**b**) axial images in a patient with pneumonia and new onset haemoptysis demonstrates a blush of contrast within the right lower lobe consolidation concerning for active bleeding (*arrow*). Bronchial arteriogram demonstrates a blush of contrast (*arrow*) confirming bleed pre embolisation (**c**) with resolution of the contrast blush post embolisation (**d**)

Although many congenital and acquired malformations of the bronchial arteries are rare, haemoptysis secondary to bronchial artery dilatation is a frequently encountered complication. In fact, the majority of haemoptysis cases requiring intervention/embolisation occur secondary to bronchial artery pathology (Fig. 21) [53, 54]. Please note, the dilatation of the bronchial arteries in response to the disease states mentioned increases the risk of clinically significant haemoptysis, as these vessels are predisposed to rupture [51, 55]. Massive haemoptysis has an extremely high mortality rate without intervention [55]. CT angiographic imaging in the aortic phase with volumetric reformatted images is recommended for evaluation of the bronchial arteries prior to embolisation and improves outcomes [52, 56].

## Summary

In this pictorial review, we have presented a broad overview of pathology involving the pulmonary arteries, pulmonary veins and the bronchial arteries. Knowledge of these conditions, as well as normal anatomy of the pulmonary vasculature, is the cornerstone for building a differential diagnosis tailored to a patient’s clinical presentation and imaging features. This is especially important as many of these conditions have significant morbidity and mortality, underscoring the importance of the material presented.
